# Vanillin Suppresses Cell Motility by Inhibiting STAT3-Mediated HIF-1α mRNA Expression in Malignant Melanoma Cells

**DOI:** 10.3390/ijms18030532

**Published:** 2017-03-01

**Authors:** Eun-Ji Park, Yoon-Mi Lee, Taek-In Oh, Byeong Mo Kim, Beong-Ou Lim, Ji-Hong Lim

**Affiliations:** 1 Department of Biomedical Chemistry, College of Biomedical & Health Science, Konkuk University, Chungju 380-701, Korea; peunji0503@kku.ac.kr (E.-J.P.); dk1050@kku.ac.kr (T.-I.O.); beongou@kku.ac.kr (B.-O.L.); 2 Interdisciplinary Research Center for Health, Konkuk University, Chungju 380-701, Korea; yoonmilee@kku.ac.kr; 3 Severance Integrative Research Institute for Cerebral and Cardiovascular Diseases (SIRIC), Yonsei University College of Medicine, Seodamun-gu, Seoul 03722, Korea; BKIM2@yuhs.ac

**Keywords:** vanillin, HIF-1α, STAT3, migration, melanoma

## Abstract

Recent studies have shown that vanillin has anti-cancer, anti-mutagenic, and anti-metastatic activity; however, the precise molecular mechanism whereby vanillin inhibits metastasis and cancer progression is not fully elucidated. In this study, we examined whether vanillin has anti-cancer and anti-metastatic activities via inhibition of hypoxia-inducible factor-1α (HIF-1α) in A2058 and A375 human malignant melanoma cells. Immunoblotting and quantitative real time (RT)-PCR analysis revealed that vanillin down-regulates HIF-1α protein accumulation and the transcripts of HIF-1α target genes related to cancer metastasis including fibronectin 1 (*FN1*), lysyl oxidase-like 2 (*LOXL2*), and urokinase plasminogen activator receptor (*uPAR*). It was also found that vanillin significantly suppresses HIF-1α mRNA expression and de novo HIF-1α protein synthesis. To understand the suppressive mechanism of vanillin on HIF-1α expression, chromatin immunoprecipitation was performed. Consequently, it was found that vanillin causes inhibition of promoter occupancy by signal transducer and activator of transcription 3 (STAT3), but not nuclear factor-κB (NF-κB), on *HIF1A*. Furthermore, an in vitro migration assay revealed that the motility of melanoma cells stimulated by hypoxia was attenuated by vanillin treatment. In conclusion, we demonstrate that vanillin might be a potential anti-metastatic agent that suppresses metastatic gene expression and migration activity under hypoxia via the STAT3-HIF-1α signaling pathway.

## 1. Introduction

Malignant melanoma is a skin cancer that develops from the abnormal growth and differentiation of melanocytes with hyperpigmentation; the incidence of melanoma cases has been increasing, and this particular skin cancer is associated with a high rate of mortality caused by early and rapid metastasis [1]. Significant therapeutic advances have been made using small molecule inhibitors that target melanoma, but challenges to eradicate these solid tumors still persist.

Given the fact that a hypoxic microenvironment is a major feature in multiple types of solid cancers including melanoma, hypoxia-inducible factor-1 (HIF-1) composed of α and β subunits is a pivotal transcription factor in the adaptation of cells to low oxygen conditions. HIF-1α protein is tightly regulated by oxygen levels despite the constitutive expression of HIF-1α mRNA. Under normoxic conditions, HIF-1α gets degraded before it can be translocated to the nucleus, associate with HIF-1β, and begin the hypoxic response that is conducive to tumor formation. For this degradation to occur, HIF-1α is hydroxylated by the prolyl 4-hydroxylase (P4H) enzyme into proline 402 and 564 residues, which cause HIF-1α to bind to the von Hippel-Lindau protein (VHL) E3-ubiquitin ligase complex, leading to ubiquitination of this complex and subsequent signaling for proteasomal degradation. Conversely, hypoxic conditions arrest this oxygen-dependent reaction, resulting in the dimerization of α and β subunits in the nucleus. Additionally, oncogenic signaling pathways such as the PI3K (phosphoinositide 3-kinase)-AKT-mTOR (mammalian target of rapamycin )axis and mitogen-activated protein kinase (MAPK) pathway are involved in de novo HIF-1α protein synthesis via 5′-cap-dependent translation initiation [2,3], and various transcription factors including signal transducer and activator of transcription 3 (STAT3) and nuclear factor-κB (NF-κB) are critical for regulating HIF-1α mRNA expression with promoter occupancy at the proximal region of *HIF1A* [4,5,6,7]. The HIF-1α and HIF-1β complex binds to the hypoxia-response element (HRE), thereby affecting various downstream genes associated with cancer cell angiogenesis, migration, and metastasis. During cancer metastasis, HIF-1α in hypoxic microenvironments transcriptionally increases various transcripts related to stimulation of cell migration and invasion, including fibronectin 1 (*FN1*), urokinase plasminogen activator receptor (*uPAR*), lysyl oxidase-like 2 (*LOXL2*), and matrix metalloproteinases (*MMPs*) [8].

Vanillin is a major component of vanilla bean extract and is widely used as a flavoring in foods. Because of its antioxidant properties, many biological activities of vanillin have been studied. Vanillin inhibited mutagen induced-DNA damage or spontaneous mutation in bacteria and human cells by eliciting DNA repair [9,10,11]. Recent reports have shown that vanillin has anti-cancer effects through increased apoptosis and cell cycle arrest in melanoma, colon, and cervical cancer cells [12,13,14]. In addition, vanillin has also been reported to exhibit anti-invasive and anti-metastatic activities by suppressing the phosphoinositide 3-kinase (PI3K) and NF-κB signaling pathways in lung, breast, and liver cancer cells [15,16,17]. Nevertheless, the precise molecular mechanism by which vanillin suppresses cancer growth and metastatic potential has not yet been elucidated. Taking into consideration all of the above facts, this study focused on the role of vanillin in the suppression of cancer cell motility and the mechanism of HIF-1α inhibition under hypoxic environments in A2058 and A375 malignant melanoma cells. 

In the present study, we evaluated the inhibitory effects of vanillin on hypoxia-inducible factor (HIF)-1α accumulation and cancer cell motility under hypoxia by abrogating STAT3-mediated HIF1A mRNA expression. Our results suggest that vanillin is a potential therapeutic compound that can be used to develop anti-metastatic agents or preventive functional foods for malignant melanoma.

## 2. Results

### 2.1. Anti-Cancer Effects of Vanillin in Human A2058 and A375 Malignant Melanoma Cells

**Figure 1:**
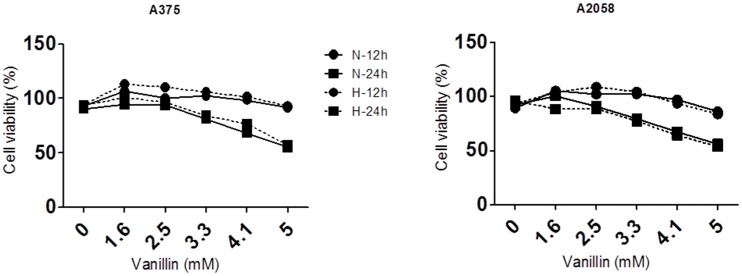
Anti-cancer effects of vanillin upon normoxia or hypoxia. Anti-cancer effects of vanillin in A375 and A2058 malignant melanoma cells. Cells were incubated with 1.6, 2.5, 3.3, 4.1, and 5.0 mM of vanillin for 12 h or 24 h under normoxic (N) or hypoxic (H) condition. Cell viability was measured by crystal violet assay as described in the materials and methods section.

Because the anti-cancer effect of vanillin under hypoxic conditions has not been previously reported, we measured cell viability under both normoxia and hypoxia in the absence or presence of vanillin. Vanillin did not have any significant effect on the viability of A2058 and A375 melanoma cells under hypoxia (Figure 1).

### 2.2. Vanillin Decreases HIF-1α Protein Levels under Hypoxia in A2058 and A375 Malignant Melanoma Cells

**Figure 2:**
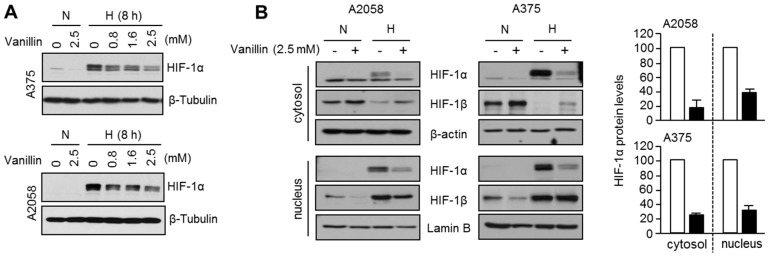
Vanillin decreases hypoxia-inducible factor (HIF)-1α protein levels upon hypoxia. (**A**) Vanillin suppresses hypoxia-induced HIF-1α protein levels. Cells were incubated in the absence or presence of vanillin for 8 h under normoxic or hypoxic condition. HIF-1α protein levels were measured by immunoblotting using anti-HIF-1α antibody; (**B**) Nuclear HIF-1α protein was decreased by vanillin treatment under hypoxia. Protein levels were quantified using Image J 1.49v software (National Institutes of Health, Bethesda, MD, USA). Values represent the mean ± standard deviations of three independent experiments performed.

We investigated the inhibitory effect of vanillin on hypoxia-induced HIF-1α accumulation. Based on our results, vanillin strongly suppressed hypoxia-induced HIF-1α accumulation, and this effect was independent of any toxicity to A2058 and A375 cells (Figure 2A). To determine whether vanillin also decreases nuclear HIF-1α protein, we measured nuclear HIF-1α protein levels under both normoxia and hypoxia in the absence or presence of vanillin. We found that vanillin inhibits HIF-1α accumulation in the nucleus (Figure 2B), suggesting that vanillin could suppress both HIF-1α transcriptional activity and HIF-1α protein levels. In addition, HIF-1β protein levels were slightly increased under vanillin treatment in the cytoplasm, but decreased in the nucleus. Because HIF-1β is translocated into the nucleus through its interaction with HIF-1α, the suppression of HIF-1α protein levels by vanillin treatment may change HIF-1β protein levels in the cytoplasm and nucleus (Figure 2B).

### 2.3. Vanillin Attenuates HIF-1α Protein Synthesis

**Figure 3:**
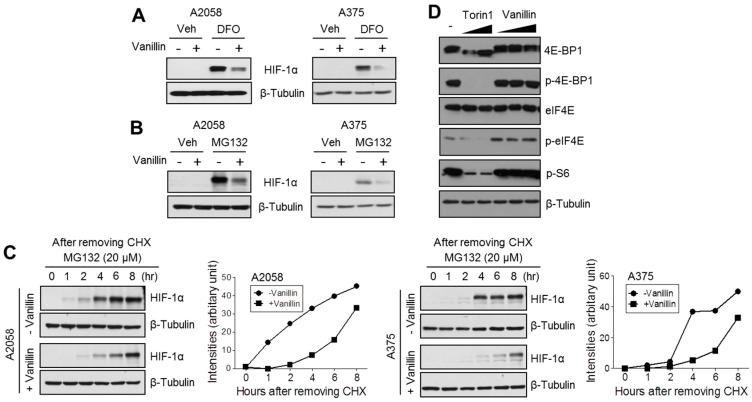
Vanillin attenuates HIF-1α protein synthesis. (**A**) Vanillin suppresses HIF-1α increased by treatment with the iron chelator, deferoxamine (DFO), under normoxic condition. A2058 and A375 cells were pre-incubated with 2.5 mM of vanillin for 1 h, and then further incubated in the absence or presence of 50 μM of DFO for 6 h; (**B**) Vanillin suppresses HIF-1α increased by proteasome inhibitor (MG132) treatment. Cells were pre-incubated with 2.5 mM of vanillin for 1 h, and then further incubated in the absence or presence of 20 μM of MG132 for 6 h; (**C**) Vanillin attenuates de novo HIF-1α protein synthesis. Cells were pre-incubated with 100 μM cycloheximide (CHX) for 12 h and then washed with phosphate-buffered saline (PBS). Cells were incubated with fresh culture medium containing 20 μM of MG132 and 2.5 mM of vanillin (or dimethyl sulfoxide (DMSO)) for indicated time. HIF-1α protein levels were detected by immunoblotting, and then protein levels were quantified using Image J software; (**D**) Vanillin does not regulate the signal transduction pathway related to 5′-cap-dependent translation.

To understand the suppression mechanism of vanillin on HIF-1α accumulation, we investigated whether the presence of vanillin could decrease HIF-1α whose increased levels were induced by using an iron chelator and 26S proteasome inhibition. Consequently, increased HIF-1α expression by both iron chelator (Figure 3A) and proteasome inhibitor (Figure 3B), as observed in the control cells, was also dramatically decreased by vanillin treatment, suggesting that neither hydroxylation nor proteasomal degradation of HIF-1α is associated with the suppressive effect of vanillin on HIF-1α accumulation. Therefore, we next investigated whether vanillin could attenuate de novo protein synthesis of HIF-1α. The results, shown in Figure 3C, demonstrate that vanillin strongly attenuates HIF-1α accumulation increased by MG132 (which blocks all proteolytic activity of the 26S proteasome complex) after blocking the de novo synthesis of HIF-1α by using CHX, as protein translation inhibitor, in both A2058 and A375 melanoma cells, suggesting that vanillin causes inhibition of de novo HIF-1α protein synthesis. Because the mammalian target of rapamycin (mTOR)-mediated phosphorylation of eukaryotic translation initiation factor 4E-binding protein 1 (4E-BP1), eukaryotic translation initiation factor 4E (eIF4E), and S6 kinase ribosomal protein stimulate de novo HIF-1α protein synthesis [2,3], we further elucidated the phosphorylation status of 4E-BP1, eIF4E, and S6 kinase after vanillin treatment. From Figure 3D, it was observed that vanillin does not alter phosphorylation of 4E-BP1, eIF4E, and S6 kinase, suggesting that mTOR-mediated de novo HIF-1α protein synthesis is not responsible for the reduction of HIF-1α by vanillin.

### 2.4. Vanillin Decreases HIF-1α mRNA Levels by Inhibiting Promoter Occupancy of STAT3 at HIF1A

**Figure 4:**
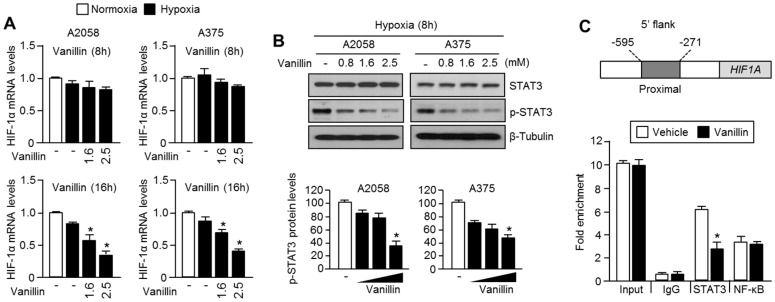
Vanillin decreases HIF-1α mRNA expression by inhibiting signal transducer and activator of transcription 3 (STAT3). (**A**) Vanillin decreases HIF-1α mRNA levels in A2058 and A375 human melanoma cells. Cells were incubated in the absence or presence of vanillin for 8 h or 16 h under hypoxic condition. HIF-1α mRNA levels were measured using quantitative real time (RT)-PCR. Values represent the mean ± SD of three independent experiments performed in duplicate; * *p* < 0.05; (**B**) Vanillin decreases STAT3 phosphorylation under hypoxia. A2058 and A375 melanoma cells were incubated with vanillin for 8 h under hypoxia. STAT3 protein levels were detected by immunoblotting, and then protein levels were quantified using Image J software. Values represent the mean ± SD of three independent experiments performed; * *p* < 0.05; (**C**) Vanillin causes dissociation of STAT3 from the HIF-1α promoter region. A2058 cells were incubated with 2.5 mM of vanillin for 8 h and then fixed with formalin. Chromatin was immunoprecipitated with non-immunized serum (IgG) or the antisera as indicated. The proximal region of HIF-1α promoter was amplified using quantitative PCR. Values represent the mean ± SD of two independent experiments performed in triplicate; * *p* < 0.05.

Because decreased mRNA levels also cause retardation of de novo protein synthesis, we next measured the HIF-1α mRNA levels after vanillin treatment of A2058 and A375 melanoma cells. We found that the HIF-1α mRNA levels were significantly decreased by vanillin treatment (Figure 4A). It is becoming clear that STAT3 and NF-κB are critical transcription factors for HIF-1α mRNA expression through direct interaction with the proximal promoter of *HIF1A* [5,6,7]. Therefore, we investigated the suppressive effect of vanillin on STAT3 activation and its promoter occupancy on *HIF1A*. Vanillin significantly decreases STAT3 phosphorylation in both A2058 and A375 melanoma cells (Figure 4B). In addition, it was also found that the proximal promoter of *HIF1A* is dissociated from STAT3, but not from NF-κB (Figure 4C). These results suggest that the inactivation of STAT3 is responsible for the decreased HIF-1α levels in response to treatment with vanillin.

### 2.5. Vanillin Down-Regulates HIF-1α Target Gene Expression and Causes Suppression of Cell Motility

**Figure 5:**
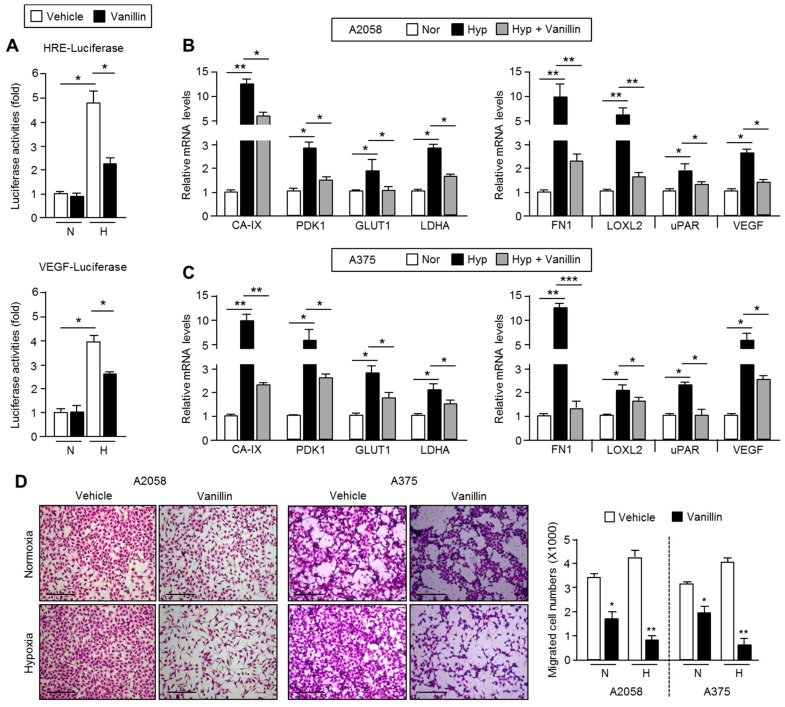
Vanillin inhibits HIF-1α transcriptional activity and cell motility in malignant melanoma cells. (**A**) Inhibitory effect of vanillin on HIF-1α transcriptional activity. A2058 cells were transiently transfected with the hypoxia-responsive element (HRE) or vascular endothelial growth factor (VEGF)–luciferase vector and then incubated for 24 h. Transfected cells were incubated under normoxia or hypoxia in the absence or presence of vanillin (2.5 mM) for 24 h. Values represent the mean ± standard deviation of three independent experiments performed in duplicate; * *p* < 0.05; (**B**,**C**) Vanillin suppresses hypoxia-induced HIF-1α target gene expression. A2058 and A375 cells were incubated under normoxia or hypoxia in the absence or presence of vanillin (2.5 mM) for 24 h. HIF-1α target gene expression was measured using quantitative RT-PCR. Values represent the mean ± standard deviation of two independent experiments performed in triplicate; * *p* < 0.05, ** *p* < 0.01, and *** *p* < 0.001; (**D**) Inhibitory effect of vanillin on cell migration. A2058 and A375 cells were seeded into transwell chambers and incubated under normoxia or hypoxia for 16 h in the absence or presence of vanillin (2.5 mM). Scale bar representing 200 μm. Migrated cell numbers were counted and values represent the mean ± standard deviation of two independent experiments performed in triplicate; * *p* < 0.05 and ** *p* < 0.01.

To determine whether vanillin functionally suppresses HIF-1α transcriptional activity as well as protein levels, HIF-1α responsive promoter activity (HRE- or vascular endothelial growth factor (VEGF)-luciferase) was measured. In this case, vanillin dramatically down-regulates HIF-1α promoter activity (Figure 5A) and its target genes involved in glycolytic metabolism (*CA-IX*, *PDK1*, *GLUT1*, and *LDHA*: carbonic anhydrase 9, pyruvate dehydrogenase kinase 1, glucose transporter 1, and lactate dehydrogenase A) and cancer metastasis (*FN1*, *LOXL2*, and *uPAR*) under hypoxia in A2058 and A375 cells (Figure 5B,C). Because HIF-1α stimulates cancer cell motility and invasiveness under hypoxia [8], the inhibitory effect of vanillin on cell migration increased by hypoxia was tested. Consequently, it was found that vanillin strongly attenuates cell migration under hypoxia in A2058 and A375 cells (Figure 5D). These results demonstrate that vanillin suppresses cancer cell motility by inhibiting HIF-1α target gene expression associated with cancer metastasis.

## 3. Discussion

Because intratumoral hypoxia causes HIF-1α overexpression, genetic alterations of HIF-1α are commonly observed in malignant solid cancers and closely associated with treatment failure and increased mortality; therefore, it is important to identify HIF-1α inhibitors and test their efficacy as anticancer therapeutics [8]. A growing number of HIF-1α inhibitors derived from natural products, low molecular weight secondary metabolites produced by plants and microbes, have recently been identified as HIF-1α inhibitors [18]. For example, it has been reported that apigenin (4′,5,7-trihydroxyflavone) and resveratrol (*trans*-3,4,5′-trihydroxystilbene) promote HIF-1α protein degradation in a manner that is independent of the microenvironment oxygen levels [19,20]. Pleurotin and genistein (4′,5,7-Trihydroxyisoflavone) inhibit the accumulation of HIF-1α protein by suppressing protein synthesis under both normoxic and hypoxic conditions [21,22]. Many of the currently identified HIF-1α inhibitors derived from natural products affect protein accumulation or degradation. Interestingly, we propose that vanillin may be a promising HIF-1α inhibitor that acts to reduce HIF-1α levels by suppressing HIF-1α mRNA expression.

Vanillin, a widely used flavoring agent from vanilla, has been shown to exhibit multiple biological effects, including anti-cancer, anti-mutagenic, and anti-bacterial activity in mammalian cells [9,14,23]. In this study, we demonstrate that vanillin effectively decreases HIF-1α protein levels and the expression of its target genes related to cell motility, angiogenesis, and glycolytic metabolism in A2058 and A375 malignant melanoma cells.

To understand the precise molecular mechanism by which vanillin decreases HIF-1α protein levels, we investigated whether vanillin attenuates HIF-1α protein synthesis or promotes proteasomal degradation. We found that vanillin dramatically attenuates de novo HIF-1α protein synthesis. In addition, MG132, a 26S proteasome inhibitor, did not rescue vanillin-mediated HIF-1α reduction in melanoma cells, suggesting that vanillin reduces HIF-1α using a mechanism that is independent of proteasomal degradation. Because the growth factor-mediated PI3K-mTOR signaling pathway activates HIF-1α protein synthesis via 4E-BP1, eIF4E, and S6 kinase linked 5′-cap-dependent translation initiation [2,3], we tested whether vanillin regulates the phosphorylation status of 4E-BP1, eIF4E, and S6 kinase using torin1, a selective mTOR inhibitor, as a positive control. Unlike torin1, vanillin did not alter the phosphorylation status of 4E-BP1, eIF4E, and S6 kinase, suggesting that vanillin does not participate in PI3K-mTOR-mediated protein synthesis. Therefore, we further investigated the inhibitory effect of vanillin on HIF-1α mRNA expression. Interestingly, HIF-1α mRNA was significantly decreased by vanillin treatment in A2058 melanoma cells.

How does vanillin decrease HIF-1α mRNA expression? To answer this question, we investigated the inhibitory effect of vanillin on STAT3-mediated HIF-1α mRNA expression, because STAT3 is one of the transcription factors that is associated with the proximal promoter of *HIF1A* [5,6]. Vanillin reduces STAT3 phosphorylation and promoter occupancy on the 5′-flank of *HIF1A*. These results suggest that vanillin decreases HIF-1α by suppressing STAT3-mediated transcription. Nevertheless, we did not provide a precise molecular mechanism to explain how vanillin inhibits STAT3 phosphorylation and proximal promoter occupancy on the 5′ flanking region of *HIF1A*. Therefore, how vanillin suppresses STAT3 phosphorylation and transcriptional activity should be further investigated.

Although the anti-metastatic effect of vanillin by inhibiting MMP-9 expression in breast and hepatocellular carcinoma cells has recently been reported, the molecular mechanism by which vanillin attenuates migration and invasion in cancer cells is not fully demonstrated [15,16,17]. In the present study, we provide insight into some part of the mechanism that involves the STAT3-HIF-1α axis on the vanillin-mediated suppression of cancer cell migration and invasion. Indeed, cell migration was significantly decreased by vanillin by approximately 50% under normoxic condition. Under hypoxia, vanillin suppressed cell migration by approximately 75%, suggesting that vanillin could sensitively block cell motility in malignant tumors with hypoxic microenvironments.

On the basis of previous studies of the anti-metastatic effects of millimolar-range vanillin used to treat cells in vivo, mice were administered 100 mg/kg/day vanillin. Although 100 mg/kg/day can be regarded as a high concentration, no side effects were observed [17]. Variable concentrations below 100 mg/kg/day can be considered for animal studies. Several methods can be used to determine the effects low-concentration vanillin. Vanillin derivatives can be developed to improve delivery efficacy at low concentrations, which are more effective for preventing malignant melanoma metastasis. Indeed, a recent report showed that 60 mg/kg/day of *o*-vanillin, a vanillin isomer, strongly suppressed tumor growth in mice bearing A375 human malignant melanoma xenografts [14]. For clinical application, it should be further evaluated whether *o*-vanillin has anti-metastatic effects via the inhibition of HIF-1α accumulation. In addition, vanillin may be useful as a functional food and not limited to chemotherapy. Our results provide a foundation for further analysis of vanillin for the prevention and treatment of malignant melanoma. 

## 4. Materials and Methods

### 4.1. Reagents and Antibodies

Vanillin (V1104), deferoxamine (D9533), MG132 (M7449), dimethyl sulfoxide (DMSO), protease inhibitor cocktail, and cycloheximide (01810) were purchased from Sigma-Aldrich (St. Louis, MO, USA). Antibodies against 4E-BP1 (9452), phospho-4E-BP1 (2855), eIF4E (9742), phospho-eIF4E (9741), phospho-S6 (4857), STAT3 (12640), and phospho-STAT3 (9131) were obtained from Cell Signaling Technology (Danvers, MA, USA). Antibodies against β-tubulin (sc-9104) and β-actin (sc-47778) were purchased from Santa Cruz Biotechnology (Santa Cruz, CA, USA). Anti-HIF-1α and anti-HIF-1β antibodies were kindly provided by Jong-Wan Park of Seoul National University, Seoul, Korea. 

### 4.2. Cell Culture and Treatment 

A2058 and A375 melanoma cell lines were purchased from American Type Culture Collection (ATCC, Manassas, VA, USA). Cells were cultured in Dulbecco’s modified Eagle’s medium (DMEM) containing 10% fetal bovine serum (FBS) (Gibco, Carlsbad, CA, USA) and 25 mM glucose in a humidified atmosphere of 5% CO_2_ at 37 °C. The oxygen level in the hypoxia incubator chamber was maintained at 1% by continuously injecting N_2_ gas. Vanillin at various stock concentrations (0.8, 1.6, 2.5 M) was dissolved in dimethyl sulfoxide (DMSO) and diluted into culture medium prior to treatment. 

### 4.3. Cell Viability Assay

To determine the cell viability, we used crystal violet staining in this study. Cells were seeded in 24-well plates, and incubated for 24 h, followed by treatment with vanillin in increasing concentrations for 12 and 24 h at 20% (normoxia) or 1% (hypoxia) oxygen conditions. The cells were fixed with 4% paraformaldehyde for 15 min and stained with 0.05% crystal violet staining solution (HT90132, Sigma-Aldrich) for 15 min. To measure optical density, 1% sodium dodecyl sulfate (SDS) solution was added to the cells, and these were further incubated for 15 min at room temperature. The dissolved solutions were transferred to a 96-well plate and measured at 595 nm using a microplate reader (BioTek, Winooski, VT, USA).

### 4.4. Cytosolic and Nuclear Extract Preparation

Cells were washed using cold phosphate-buffered saline and harvested by centrifugation at 1000 rpm for 5 min at 4 °C. The harvested cell pellets were resuspended and incubated with ice-cold buffer A (20 mM Tris at pH 7.8, 1.5 mM MgCl_2_, 10 mM KCl, 0.2 mM ethylenediaminetetraacetic acid (EDTA), 0.5 mM dithiothreitol (DTT), and protease inhibitor cocktail) for 5 min on ice, and then cells were collected by centrifugation at 1000 rpm for 5 min at 4 °C. Next, the cell pellets were lysed using 0.06% NP-40 containing buffer A for 10 min on ice. The cell lysates were centrifuged at 3000 rpm for 5 min, and then the supernatants containing cytosolic proteins were frozen. After obtaining the cytosolic fraction, the pellets were incubated and lysed using buffer B (20 mM Tris-Cl at pH 7.8, 1.5 mM MgCl_2_, 0.2 mM EDTA, 0.5 mM DTT, and 20% glycerol) containing 400 mM NaCl for 30 min on ice. During incubation, the cells were homogenized with a glass homogenizer. The incubated samples were centrifuged at 14,000 rpm for 30 min at 4 °C, and then supernatants containing the nuclear proteins were transferred into fresh tubes. 

### 4.5. Immunoblotting

Total proteins were extracted using cell lysis buffer (1% NP-40, 150 mM NaCl, 50 mM Tris pH 7.4, 2 mM EDTA, and protease inhibitor cocktail). Cell lysates were separated by 7.5% or 10% SDS-polyacrylamide gel electrophoresis (PAGE). Separated proteins were transferred onto an Immobilon-P membrane (Millipore, Billerica, MA, USA). The transferred membranes were blocked with 5% skim milk in Tris-buffered saline containing 0.05% Tween-20 (TBS-T) for 1 h at room temperature, and then incubated overnight with primary antibodies diluted at 1:1000 or 1:5000 in 5% skim milk in TBS-T at 4 °C. Horseradish peroxidase-conjugated secondary antibodies were incubated for 1 h at room temperature, and then protein levels were visualized using an Enhanced Chemiluminescence Prime kit (GE Healthcare, Little Chalfont, UK). 

### 4.6. Quantitative Real-Time PCR

Total RNA was isolated with TRIzol and 2 μg of this RNA was used to synthesize cDNA using a high capacity cDNA reverse transcription kit (Applied Biosystems). The cDNA was amplified over 40 cycles (95 °C for 15 s, 60 °C for 1 min). Experimental *C*_q_ values were normalized to *H36B4* and relative mRNA levels were calculated on the basis of H36B4 mRNA levels. The sequences of the PCR primers (5′–3′) were: ATGGAGCCCAGCAGCAA and GGCATTGATGACTCCAGTGTT for *GLUT1*; CCACTCCAGCAGGGAAGG and GCGACGCAGCCTTTGAAT for *CA-IX*; TGAACATTCTGGCTGGTGACAGGA and ATGATGTCATTCCCACAATGGCCC for *PDK1*; CTACCTCCACCATGCCAAGT and AGCTGCGCTGATAGACATCC for *VEGF*; CCATAAAGGGCAACCAAGAG and ACCTCGGTGTTGTAAGGTGG for *FN1*; CACTGCGGATCCCTGAAAC and CCTGTCTTCGGGCTGATG for *LOXL2*; AGCCTTACCGAGGTTGTGTG and AAATGCATTCGAGGTAACGG for *uPAR*.

### 4.7. In Vitro Migration Assay

In vitro cell migration assays were performed using a Transwell chamber from Sigma-Aldrich (St. Louis, MO, USA). The underside of the Transwell insert membrane was coated with collagen and incubated at room temperature until it was dry. Cells in 0.1 mL of fetal bovine serum-free medium were seeded into the upper chamber, and the lower chamber was filled with 6% fetal bovine serum-containing medium as a chemotactic source, and then cells were incubated for 16 h at 37 °C. After incubation, the Transwell chambers were quickly washed using phosphate-buffered saline and stained with hematoxylin and eosin. The Transwell insert membranes, containing the migrated cells, were placed on a slide glass and analyzed using a microscope (Olympus, Tokyo, Japan). To quantify the migrated cell numbers, two random fields under 40× magnification were quantified by counting the cell numbers.

### 4.8. Chromatin Immunoprecipitation

Cultured cells were fixed with 1% formaldehyde to cross-link chromatin and proteins, and soluble chromatin and protein complex samples were incubated overnight at 4 °C with antibodies against STAT3 and NF-κB p65. STAT3 or NF-κB p65 interacting DNA was eluted, and then occupancy of STAT3 on the proximal region of the HIF-1α promoter was measured using quantitative PCR. The sequences of PCR primers for the quantitative ChIP assay (5′–3′) are ATCTGAGCAACGAGACCAAA and CACGTGCTCGTCTGTGTTTA.

### 4.9. Luciferase Activity Assay

Hypoxia-responsive element (HRE) or VEGF (vascular endothelial growth factor) promoter-luciferase reporter plasmids were a gift from Navdeep Chandel (Addgene plasmid # 26731) and Jong-Wan Park (Seoul National University, Korea). Cells were transfected with reporter plasmids using Polyfect (QIAGEN, Valencia, CA, USA) and then incubated for 48 h for stabilization. After incubation, luciferase activity was measured using a luminometer (Berthold Technologies, Bad Wildbad, Germany) and normalized against β-gal activities to account for transfection efficiency.

### 4.10. Statistical Analysis

All data were analyzed using an unpaired Student’s *t*-test for two experimental comparisons and a one-way ANOVA (analysis of variance) followed by Tukey’s post hoc test for multiple comparisons using GraphPad Prism 5.01 (GraphPad Software Inc., La Jolla, CA, USA). Data are represented as means ± standard deviations (SDs). Differences between mean values were considered statistically significant when the associated *p*-value was less than 0.05.

## 5. Conclusions

In the present study, the major findings were that vanillin: (i) decreases HIF-1α protein levels and that this effect is independent of proteasomal degradation; (ii) suppresses STAT3 phosphorylation and its promoter occupancy on the proximal region of HIF-1α; and (iii) attenuates cell migration by down-regulating HIF-1α target genes associated with cancer metastasis in A375 and A2058 human malignant melanoma cells. Taken together, these results may provide useful information for the development of vanillin derivatives as anti-metastatic agents or functional foods for preventing malignant melanoma metastasis.

## Abbreviations

HIF-1α	Hypoxia-inducible factor-1α
STAT3	Signal transducer and activator of transcription 3
NF-κB	Nuclear factor-κB
FN1	Fibronectin 1
LOXL2	Lysyl oxidase-like 2
uPAR	Urokinase plasminogen activator receptor
VHL	Von Hippel-Lindau protein
MAPK	Mitogen activated protein kinase
HRE	Hypoxia-response element
MMPs	Matrix metalloproteinases
PI3K	Phosphoinositide 3-kinase
mTOR	Mammalian target of rapamycin
4E-BP1	Eukaryotic translation initiation factor 4E-binding protein 1
eIF4E	Eukaryotic translation initiation factor 4E
